# Movable Surface Rotation Angle Measurement System Using IMU

**DOI:** 10.3390/s22228996

**Published:** 2022-11-21

**Authors:** Changfa Wang, Xiaowei Tu, Qi Chen, Qinghua Yang, Tao Fang

**Affiliations:** School of Mechatronic Engineering and Automation, Shanghai University, Shanghai 200444, China

**Keywords:** angle measurement, inertial measurement unit, extended Kalman filter, rotation axis direction estimation

## Abstract

In this paper, we describe a rotation angle measurement system (RAMS) based on an inertial measurement unit (IMU) developed to measure the rotation angle of a movable surface. The existing IMU-based attitude (tilt) sensor can only accurately measure the rotation angle when the rotation axis of the movable surface is perfectly aligned with the X axis or Y axis of the sensor, which is always not possible in real-world engineering. To overcome the difficulty of sensor installation and ensure measurement accuracy, first, we build a model to describe the relationship between the rotation axis and the IMU. Then, based on the built model, we propose a simple online method to estimate the direction of the rotation axis without using a complicated apparatus and a method to estimate the rotation angle using the known rotation axis based on the extended Kalman filter (EKF). Using the estimated rotation axis direction, we can effectively eliminate the influence of the mounting position on the measurement results. In addition, the zero-velocity detection (ZVD) technique is used to ensure the reliability of the rotation axis direction estimation and is used in combination with the EKF as the switching signal to adaptively adjust the noise covariance matrix. Finally, the experimental results show that the developed RAMS has a static measurement error of less than 0.05° and a dynamic measurement error of less than 1° in the range of ±180°.

## 1. Introduction

**Figure 1:**
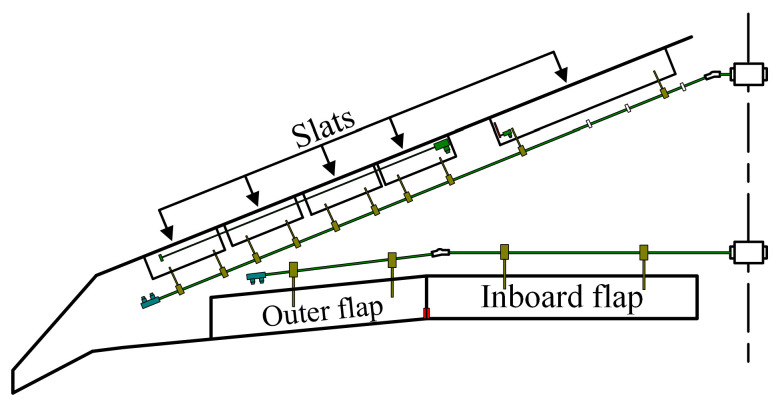
Aircraft’s movable surfaces are attached to the airframe.

The movable surfaces of an aircraft (such as elevators, flaps, and slats) are aerodynamic devices allowing a pilot to adjust and control the attitude of an aircraft. Movable surfaces are attached to the airframe on hinges or tracks (see Figure 1) and must be rotated to the angle assigned by the pilot to ensure the stability of the aircraft. Therefore, it is essential to accurately measure the rotation angles of the movable surfaces during an aircraft’s ground test.

The principle of measuring the rotation angle of a movable surface is equivalent to measuring the rotation angle of a rigid body rotating around a fixed axis, which has a specific direction. Optical motion capture systems are suitable for this task, however, these are expensive and require high computational costs [1]. Inertial measurement units (IMUs) containing tri-axis accelerometers and tri-axis gyroscopes are also suitable for this task. In recent years, microelectromechanical system (MEMS) IMUs have become widely available in many areas, such as state estimation and angle measurement, due to their small size and low cost [2,3].

Lian Hu et al. [4] proposed a Kalman filter-based algorithm that uses an IMU to estimate the roll angle of an agricultural machine in paddy fields. Milad Ghanbari et al. [5] proposed a tilt measurement algorithm consisting of a modified Kalman filter (KF) as a postfilter and a complementary filter as a prefilter to enhance the accelerometer bandwidth and eliminate the gyroscope drift. In ref. [6], the attitude (pitch and roll) was obtained by fusing the acceleration and angular velocity using a nonlinear complementary filter. In refs. [7,8,9], a KF-based algorithm using an IMU was developed to estimate the attitude during dynamic conditions. Although these methods can solve the attitude estimation problem, the pitch or roll is not equal to the rotation angle around the fixed rotation axis when neither the X nor Y axis of the IMU are aligned with the rotation axis of the movable surface.

Combining an IMU and a tri-axis magnetometer can form an attitude and heading reference system (AHRS) that can provide a complete orientation measurement relative to the direction of gravity and the Earth’s magnetic field [10,11,12]. We can parameterize the orientation measured by AHRS as two quantities: a unit vector indicating the direction of a rotation axis and an angle θ describing the magnitude of the rotation about the axis, where θ is the rotation angle of the movable surface. However, the performance of the AHRS degrades substantially due to the presence of significant magnetic disturbances in the aircraft ground test environment.

Aligning the measurement axis of the IMU with the rotation axis is hard. Estimating the mounting error (the misalignment of the IMU relative to the rotation axis) and correcting the measured pitch or roll is an effective method to measure the rotation angle. Although mounting error estimation methods can be found in numerous studies [13,14,15], these methods require an apparatus such as turntables and have to be performed in the laboratory. In ref. [16], a novel method to estimate mounting error was proposed, which requires only a tri-axis accelerometer. However, the rotation angle is obtained by solving a model equation, so the sensor noises directly affect the measurement results. Furthermore, it is impossible to measure the rotation angle while the movable surface is rotating without a gyroscope.

The primary objective of this research was to develop an IMU-based rotation angle measurement system (RAMS) that can accurately measure the rotation angle without using high-cost instruments and the RAMS can be randomly mounted on a movable surface. The measurement algorithm used in RAMS consists of two parts: rotation axis direction estimation and rotation angle estimation. The first part only requires the movable surface to be stationary for a few seconds in three different rotational positions after the RAMS is mounted on the surface while recording the accelerometer data. We can calculate the rotation axis direction in the RAMS (IMU) frame of reference from the recorded data. In the second part, an adaptive extended Kalman filter (EKF) is used to solve the rotation angle estimation problem. We use acceleration data to correct the rotation angle obtained from a gyroscope integration, where the measurement model in the EKF is built using the estimated rotation axis direction. Furthermore, the zero-velocity detection (ZVD) technique is used in both the rotation axis direction estimation and rotation angle estimation, which aims to detect whether the RAMS is stationary. The ZVD determines the moment of recording acceleration data during the rotation axis direction estimation and the moment of switching the EKF noise covariance matrix during the rotation angle estimation. The proposed algorithm is compared with the method in ref. [16], and the measurement accuracy of the RAMS is compared with a commercial high-precision AHRS (Ellipse2-N from SBG). The results show that the RAMS is able to measure the rotation angle within ±180°, and the maximum measurement error is less than 0.05°, even with it randomly mounted on a movable surface.

The rest of this article is organized as follows. Section 2 develops a model to describe the relationship between the IMU output and the rotation axis of the movable surface. Section 3 gives details of the proposed algorithm used in RAMS. Section 4 introduces the design for the RAMS. Section 5 performs a simulation to evaluate the performance of the proposed algorithm and the effect of major error sources. Section 6 presents the experimental results. Section 7 concludes this article with a summary of the designed measurement system.

## 2. Modeling

### 2.1. Problem Definition

**Figure 2:**
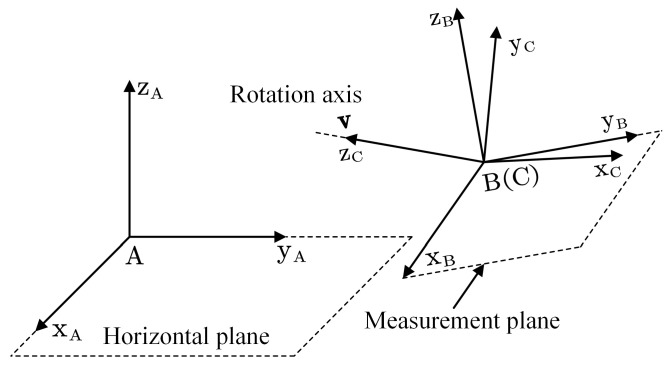
Frames of reference are used. A denotes the frame fixed on the Earth, B denotes the IMU frame of reference, and C denotes the movable surface frame of reference.

In this section, we used IMU instead of RAMS since the IMU is the core of measuring the rotation angle of a movable surface. It is required to mount the IMU on the surface to measure the rotation angle of the aircraft’s movable surface. The following notation for frames of reference is used (see Figure 2):

- A denotes the frame fixed on the Earth.
- B denotes the IMU frame of reference.
- C denotes the movable surface frame of reference.

We assume that the X–Y plane of frame A is the horizontal plane, the X–Y plane of frame B is the measurement plane, and the Z axis of frame C is the rotation axis of the movable surface.

The coordinate transformation of the vector $e\in R^{3}$ between two different frames (e.g., *A* and *B*) is

(1) $${}^{B}e={}_{B}^{A}R^{T}{}^{A}e$$

where the left superscript of e implies that the corresponding vectors are expressed in different frames, and ${}_{B}^{A}R\in R^{3\times 3}$ is the rotation matrix of the frame *B* with respect to frame *A*.

The vector ${}^{B}v={\left[\begin{array}{ccc} v_{x} & v_{y} & v_{z} \end{array}\right]}^{T}$ in frame *B* is used to represent the direction of the rotation axis with respect to the IMU, which satisfies the following conditions:

1. $\left|\right|{}^{B}v\left|\right|=\sqrt{v_{x}^{2}+v_{y}^{2}+v_{z}^{2}}=1$.
2. $v_{x}\geq 0$, and if $v_{x}=0$, $v_{y}>0$.

where ||·|| denotes the norm of the vector.

The rotation matrix R is expressed using a unit vector $⊤={\left[\begin{array}{ccc} x & y & z \end{array}\right]}^{T}$ and a rotation angle θ as [17]

(2) $$\begin{array}{cc} R & =R(\theta ,⊤)\\ & =\left(\cos\theta \right)I+\left(\sin\theta \right){\left[⊤\right]}_{\times }+\left(1-\cos\theta \right)\left(⊤{⊤}^{T}\right)\\ & \begin{array}{c} =\left[\begin{array}{cc} \cos\theta +\left(1-\cos\theta \right)x^{2} & \left(1-\cos\theta \right)xy-\left(\sin\theta \right)z\\ \left(1-\cos\theta \right)xy+\left(\sin\theta \right)z & \cos\theta +\left(1-\cos\theta \right)y^{2}\\ \left(1-\cos\theta \right)zx-\left(\sin\theta \right)y & \left(1-\cos\theta \right)zy+\left(\sin\theta \right)x \end{array}\right.\\ \left.\begin{array}{c} \left(1-\cos\theta \right)zx+\left(\sin\theta \right)y\\ \left(1-\cos\theta \right)zy-\left(\sin\theta \right)x\\ \cos\theta +\left(1-\cos\theta \right)z^{2} \end{array}\right] \end{array} \end{array}$$

where R(·) denotes the conversion of an axis-angle to a rotation matrix, and ${\left[\cdot \right]}_{\times }$ denotes conversion of a vector to a skew-symmetric matrix.

The purpose of this paper was to estimate the rotation axis ${}^{B}v$ and rotation angle θ of a movable surface.

### 2.2. Relationship between Accelerometer, Rotation Axis, and Rotation Angle

**Figure 3:**
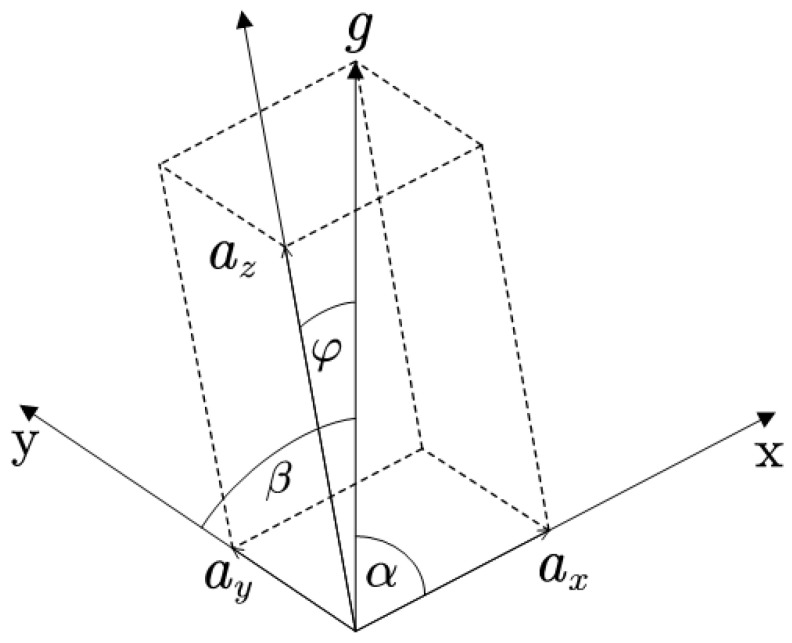
Distribution of the gravitational acceleration.

The tri-axis accelerometer can measure the external specific force acting on the sensor. When the accelerometer is stationary, the measured acceleration is the gravitational acceleration ${}^{B}g={\left[\begin{array}{ccc} a_{x} & a_{y} & a_{z} \end{array}\right]}^{T}$. The distribution of gravitational acceleration ${}^{A}g={\left[\begin{array}{ccc} 0 & 0 & g \end{array}\right]}^{T}$ on each axis as depicted in Figure 3, and the following formulas are valid [18]

(3) $$a_{x}=g\cos\alpha$$

(4) $$a_{y}=g\cos\beta$$

(5) $$a_{z}=g\cos\varphi$$

where α, β, and φ denote the angles included between gravitational acceleration and X, Y, and Z axes, respectively.

If both the measurement plane and the rotation axis are horizontal, the tilt angle can be measured in the stationary state by Equations (3)–(5). In most practical cases, however, the sensor cannot be mounted in an ideal position. The measured gravitational acceleration ${}^{B}g$ can be expressed in terms of ${}_{B}^{A}R$ as

(6) $${}^{B}g={}_{B}^{A}R^{T}{}^{A}g$$

Then, only consider the stationary state of the accelerometer, the rotation axis and the measured acceleration with respect to the frame *C* can be expressed as

(7) $${}^{C}v={}_{C}^{B}R^{T}{}^{B}v={\left[\begin{array}{ccc} 0 & 0 & 1 \end{array}\right]}^{T}$$

(8) $${}^{C}g={}_{C}^{B}R^{T}{}^{B}g$$

where ${}^{C}v$ and ${}^{C}g$ denote the rotation axis direction and the measured gravitational acceleration in frame *C*, respectively.

We assume that ${}^{C}g_{0}={\left[\begin{array}{ccc} {}^{C}a_{x_{0}} & {}^{C}a_{y_{0}} & {}^{C}a_{z_{0}} \end{array}\right]}^{T}$ denotes the measured acceleration at the initial moment when the movable surface has not yet rotated, and $\theta_{t}$ denotes the rotation angle of the movable surface at time *t*. The rotation matrix with respect to the initial moment is

(9) $$R\left(\theta_{t},{}^{C}v\right)=\left[\begin{array}{ccc} \cos\theta_{t} & -\sin\theta_{t} & 0\\ \sin\theta_{t} & \cos\theta_{t} & 0\\ 0 & 0 & 1 \end{array}\right]$$

At this moment, the measured acceleration in the frame *C* can be expressed as:

(10) $$\begin{array}{cc} {}^{C}g_{t} & =R{\left(\theta_{t},{}^{C}v\right)}^{T}{}^{C}g_{0}\\ & =\left[\begin{array}{c} {}^{C}a_{x_{0}}\cos\theta_{t}+{}^{C}a_{y_{0}}\sin\theta_{t}\\ -{}^{C}a_{x_{0}}\sin\theta_{t}+{}^{C}a_{y_{0}}\cos\theta_{t}\\ {}^{C}a_{z_{0}} \end{array}\right] \end{array}$$

**Figure 4:**
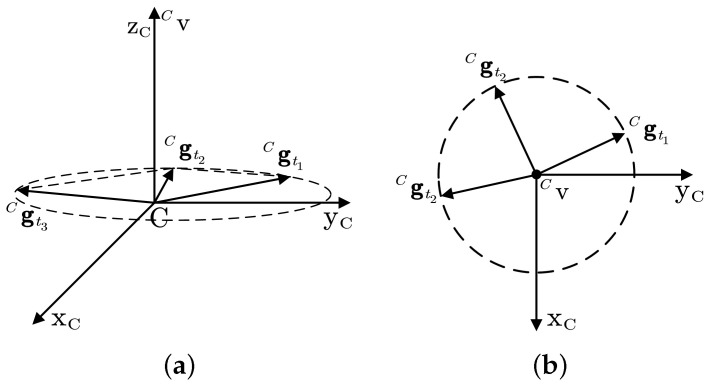
Trajectory of the measured acceleration in the frame *C*: (**a**) is the main view; and (**b**) is the top view.

We can obtain that the trajectory of ${}^{C}g_{t}$ is a circle perpendicular to ${}^{C}v$ as the rotation angle $\theta_{t}$ changes (see Figure 4).

For three different times $t_{1}$, $t_{2}$, and $t_{3}$, we can obtain

(11) $${}^{C}g_{t_{1}}=\left[\begin{array}{c} {}^{C}a_{x_{0}}\cos\theta_{t_{1}}+{}^{C}a_{y_{0}}\sin\theta_{t_{1}}\\ -{}^{C}a_{x_{0}}\sin\theta_{t_{1}}+{}^{C}a_{y_{0}}\cos\theta_{t_{1}}\\ {}^{C}a_{z_{0}} \end{array}\right]$$

(12) $${}^{C}g_{t_{2}}=\left[\begin{array}{c} {}^{C}a_{x_{0}}\cos\theta_{t_{2}}+{}^{C}a_{y_{0}}\sin\theta_{t_{2}}\\ -{}^{C}a_{x_{0}}\sin\theta_{t_{2}}+{}^{C}a_{y_{0}}\cos\theta_{t_{2}}\\ {}^{C}a_{z_{0}} \end{array}\right]$$

(13) $${}^{C}g_{t_{3}}=\left[\begin{array}{c} {}^{C}a_{x_{0}}\cos\theta_{t_{3}}+{}^{C}a_{y_{0}}\sin\theta_{t_{3}}\\ -{}^{C}a_{x_{0}}\sin\theta_{t_{3}}+{}^{C}a_{y_{0}}\cos\theta_{t_{3}}\\ {}^{C}a_{z_{0}} \end{array}\right]$$

Considering Equations (11)–(13) together, we can yield the following relationship:

(14) $${}^{C}v=\pm \frac{{\left[{}^{C}g_{t_{2}}-{}^{C}g_{t_{1}}\right]}_{\times }\left[{}^{C}g_{t_{3}}-{}^{C}g_{t_{2}}\right]}{\left|\right|{\left[{}^{C}g_{t_{2}}-{}^{C}g_{t_{1}}\right]}_{\times }\left[{}^{C}g_{t_{3}}-{}^{C}g_{t_{2}}\right]\left|\right|}$$

Multiplying both sides of Equation (14) by ${}_{C}^{B}R$ yields

(15) $${}^{B}v=\pm \frac{{\left[{}^{B}g_{t_{2}}-{}^{B}g_{t_{1}}\right]}_{\times }\left[{}^{B}g_{t_{3}}-{}^{B}g_{t_{2}}\right]}{\left|\right|{\left[{}^{B}g_{t_{2}}-{}^{B}g_{t_{1}}\right]}_{\times }\left[{}^{B}g_{t_{3}}-{}^{B}g_{t_{2}}\right]\left|\right|}$$

Therefore, we can conclude that it is only needed to measure the gravitational acceleration at three different rotation positions to calculate the rotation axis direction ${}^{B}v$.

When the external acceleration is not considered (the accelerometer is stationary), the relationship between the measured acceleration ${}^{B}a_{m}$ and the rotation angle θ can be expressed as

(16) $${}^{B}a_{m}=R{\left(\theta ,{}^{B}v\right)}^{T}{}^{B}{\bar{a}}_{0}+n_{a}$$

where ${}^{B}{\bar{a}}_{0}={\left[\begin{array}{ccc} {\bar{a}}_{x_{0}} & {\bar{a}}_{y_{0}} & {\bar{a}}_{z_{0}} \end{array}\right]}^{T}$ is the mean value of the acceleration measurements over an initialization period with no motion, and $n_{a}$ denotes the measurement noise, assumed to be Gaussian white noise, $n_{a}∼N\left(0,\sigma_{a}^{2}I_{3\times 3}\right)$.

### 2.3. Relationship between Gyroscope, Rotation Axis, and Rotation Angle

As part of the IMU, the tri-axis gyroscope provides measurements of the angular velocity about the X, Y, and Z axes. Gyroscopes are known to be affected by different error terms, such as a measurement noise error and a bias [19]. The relationship between the measured angular velocity ${}^{B}\omega_{m}$ and the real angular velocity ${}^{B}{\hat{\omega }}_{m}$ can be simply modeled as [8,9]

(17) $${}^{B}\omega_{m}={}^{B}\hat{\omega }+b+n_{\omega }$$

(18) $$\dot{b}=w$$

where b denotes the gyroscope bias, $n_{\omega }$ denotes the measurement noise, and w denotes a random walk process. Both $n_{\omega }$ and w are assumed to be Gaussian white noise, $n_{\omega }∼N\left(0,\sigma_{\theta }^{2}I_{3\times 3}\right)$, $w∼N\left(0,\sigma_{b}^{2}I_{3\times 3}\right)$.

With a fixed rotation axis, the relationship between rotation velocity, rotation axis, and rotation angle can be expressed as

(19) $${}^{B}\hat{\omega }=\pm \left|\right|{}^{B}\hat{\omega }\left|\right|{}^{B}v$$

(20) $$\dot{\theta }={}^{B}v^{T}{}^{B}\hat{\omega }={}^{B}v^{T}\left({}^{B}\omega_{m}-b+n_{\omega }\right)$$

## 3. Algorithm Description

**Figure 5:**
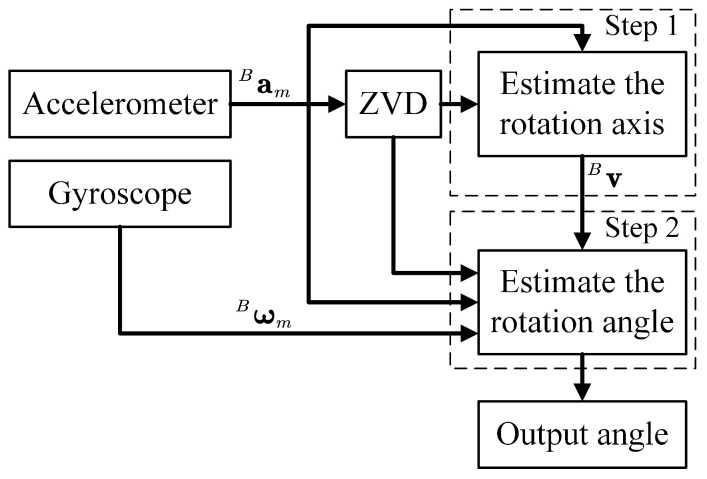
Block diagram for the angle measurement algorithm. Estimating the rotation axis direction requires acceleration data and the results of zero-velocity detection (ZVD). Estimating the rotation angle requires acceleration data, angular velocity data, rotation axis direction, and the results of ZVD.

The whole angle measurement process is performed in two steps. The first step is to estimate the rotation axis direction ${}^{B}v$. The second step is using ${}^{B}v$ to construct the prediction model and the measurement model in the EKF, which is used to estimate the rotation angle. In addition, the ZVD is required for both rotation axis direction estimation and rotation angle estimation. Figure 5 shows the block diagram of the angle measurement algorithm. More details about the algorithm are introduced as follows.

### 3.1. Zero-Velocity Detection

The objective of ZVD is to decide whether the IMU is stationary or moving during a time epoch consisting of *N* measurements between time instants n and n+N−1 [20]. The ZVD is widely used in inertial navigation systems, providing the required information to reset the velocity error, and preventing the velocity error linearly increasing with time [21,22]. When the IMU is stationary, the following two conditions should be satisfied [23]:

1. Acceleration condition: $\left|\right|{}^{B}a_{m}\left|\right|=g$.
2. Angular velocity condition: $\left|\right|{}^{B}\omega_{m}\left|\right|=0$.

Isaac Skog et al. [20] confirmed that a simple threshold on the angular velocity magnitude works well. Antonio R. Jiménez et al. [24] applied thresholds to the acceleration magnitude, angular velocity magnitude, and local acceleration variance. In ref. [25], a time duration threshold was added. In this paper, the ZVD conditions are:

1. The acceleration magnitude needs to satisfy a threshold:
  (21) $$th_{a_{\min}}<||{}^{B}a_{m}\left|\right|<th_{a_{\max}}.$$
2. The local acceleration variance is less than a threshold:
  (22) $$\zeta_{a}^{2}={\mathrm{var}}_{t_{w}}\left(\left|\right|{}^{B}a_{m_{t}}\left|\right|\right)<th_{\zeta_{a}}$$
  where ${\mathrm{var}}_{t_{w}}\left(|\right|{}^{B}a_{m_{t}}\left||\right)$ is an operator that computes the variance of acceleration magnitude $\left|\right|{}^{B}a_{m_{t}}\left|\right|$ measured in a time interval of length $t_{w}$ seconds.
3. Both of the above conditions need to be satisfied, and the duration is longer than $th_{t}$ seconds.

**Figure 6:**
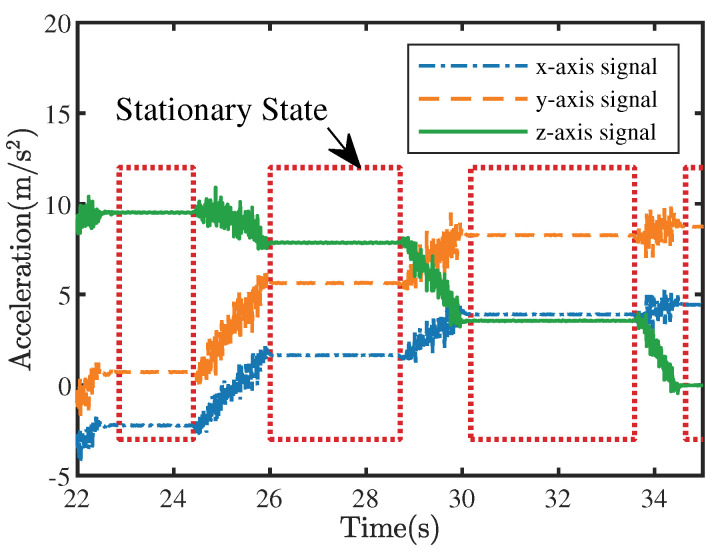
An example of the zero-velocity detection applied to the acceleration data.

Figure 6 describes an example of how the ZVD works on actual acceleration data, and Table 1 shows the parameter settings at this point.

### 3.2. Estimation of Rotation Axis Direction

After mounting the RAMS on a movable surface, the first step is to estimate the rotation axis direction in the frame *B*. As introduced in Section 2, the key of the rotation axis direction estimation is to measure the gravitational acceleration at three different rotational positions, and then the rotation axis direction ${}^{B}v$ can be calculated from Equation (15).

We average the acceleration data when the movable surface is stationary after being rotated to mitigate the measurement noise effect on the result of Equation (15), and rewrite Equation (15) as

(23) $${}^{B}v=\pm \frac{{\left[{}^{B}{\bar{a}}_{1}-{}^{B}{\bar{a}}_{0}\right]}_{\times }\left[{}^{B}{\bar{a}}_{2}-{}^{B}{\bar{a}}_{1}\right]}{\left|\right|{\left[{}^{B}{\bar{a}}_{1}-{}^{B}{\bar{a}}_{0}\right]}_{\times }\left[{}^{B}{\bar{a}}_{2}-{}^{B}{\bar{a}}_{1}\right]\left|\right|}$$

where ${}^{B}{\bar{a}}_{0}$, ${}^{B}{\bar{a}}_{1}$, and ${}^{B}{\bar{a}}_{2}$ denote the average acceleration.

The ZVD ensures that stationary and motion intervals are correctly classified. Once the gravitational acceleration measurements at three different positions are obtained, the rotation axis direction ${}^{B}v$ can be estimated according to Equation (23).

The operation procedure rotates the movable surface and then keeps it stationary for at least 1 s, while recording the acceleration data and inputting them into Algorithm A1 (see Appendix A). This is repeated operation until Algorithm A1 successfully obtains the rotation axis direction ${}^{B}v$.

### 3.3. Estimation of Rotation Angle

An EKF, which consists of two stages (prediction and correction) [7,8,9,10,12], is used to estimate the rotation angle after the rotation axis direction is estimated. We define the rotation angle $\theta_{k}$ and the gyroscope bias $b_{k}$ as state vectors

(24) $$x_{k}=\left[\begin{array}{c} \theta_{k}\\ b_{k} \end{array}\right].$$

#### 3.3.1. Prediction

Assume that at time step *k*, we have the angular velocity ${}^{B}\omega_{m}$ measured by gyroscope and the rotation axis direction ${}^{B}v$. According to Equations (17)–(20), we can obtain the prediction equation of the EKF as

(25) $$\begin{array}{cc} {\hat{x}}_{k}^{-} & =f({\hat{x}}_{k-1},{}^{B}\omega_{m})\\ & =\left[\begin{array}{c} {\hat{\theta }}_{k-1}+{}^{B}v^{T}\hat{\omega }\Delta t\\ {\hat{b}}_{k-1} \end{array}\right] \end{array}$$

(26) $$P_{k}^{-}=F_{x}P_{k-1}F_{x}^{T}+Q$$

where $\hat{\omega }={}^{B}\omega_{m}-{\hat{b}}_{k-1}$, the minus superscript denotes the a priori (or predicted) estimate, the hat represents that the real system state is estimated by the EKF, $P_{k-1}$ is the covariance matrix, $F_{x}$ is the Jacobi matrix of $f({\hat{x}}_{k|k},\omega_{m})$, and Q is the process noise covariance matrix.

The process noise covariance matrix Q is

(27) $$Q=B\left[\begin{array}{cc} \sigma_{\theta }^{2}I_{3\times 3} & 0\\ 0 & \sigma_{b}^{2}I_{3\times 3} \end{array}\right]B^{T}$$

(28) $$B=\left[\begin{array}{cc} {}^{B}v^{T}\Delta t & 0\\ 0 & \Delta tI_{3\times 3} \end{array}\right]$$

where $\sigma_{\theta }^{2}I_{3\times 3}$ and $\sigma_{b}^{2}I_{3\times 3}$ are the covariance matrix of $n_{\omega }$ and w.

#### 3.3.2. Correction

We use the measured acceleration ${}^{B}a_{m}$ to correct the prior estimate. According to Equation (16), the correction model is

(29) $${}^{B}a_{m}=h\left({\hat{x}}_{k}^{-}\right)+n_{a}=R{\left(\theta ,{}^{B}v\right)}^{T}{}^{B}{\bar{a}}_{0}+n_{a}$$

where ${}^{B}{\bar{a}}_{0}$ and $n_{a}$ are defined in the same way as in Equation (16).

The prediction equation can be written as

(30) $${\hat{x}}_{k}={\hat{x}}_{k}^{-}+K\left({}^{B}a_{m}-h\left({\hat{x}}_{k}^{-}\right)\right)$$

(31) $$K=P_{k}^{-}{H_{x}}^{T}{\left(H_{x}P_{k}^{-}{H_{x}}^{T}+V\right)}^{-1}$$

(32) $$P_{k}=\left(I-{\mathrm{KH}}_{x}\right)P_{k}^{-}$$

where $H_{x}$ is the Jacobi matrix of $h({\hat{x}}_{k}^{-})$, K is Kalman gain, and V is the measurement noise covariance matrix.

The measurement noise covariance matrix V is

(33) $$V=\sigma_{a}^{2}I_{3\times 3}$$

where $\sigma_{a}^{2}I_{3\times 3}$ is the covariance matrix of $n_{a}$.

**Figure 7:**
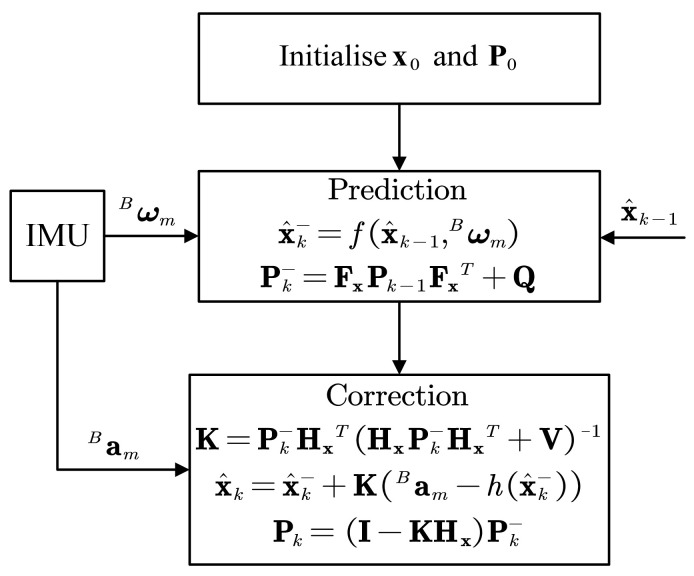
Procedure of the EKF to estimate the rotation angle.

The procedure of the EKF to estimate the rotation angle is shown in Figure 7.

#### 3.3.3. Dynamic Adjustment of Noise Covariance

The above measurement model is only valid when the RAMS is stationary due to the external acceleration. Methods to resolve the covariance uncertainty due to external acceleration by adaptively adjusting the measurement noise covariance matrix can be found in many studies [7,8,9,26]. We chose a switching EKF structure to eliminate the effect of external acceleration.

Based on the standard EKF, we define two state covariance matrices P and $P_{z}$, and two measurement noise covariance matrices V and $V_{z}$. $P_{z}$ and $V_{z}$ are used to construct the EKF for stationary intervals. P and V are used to construct the EKF for motion intervals.

**Figure 8:**
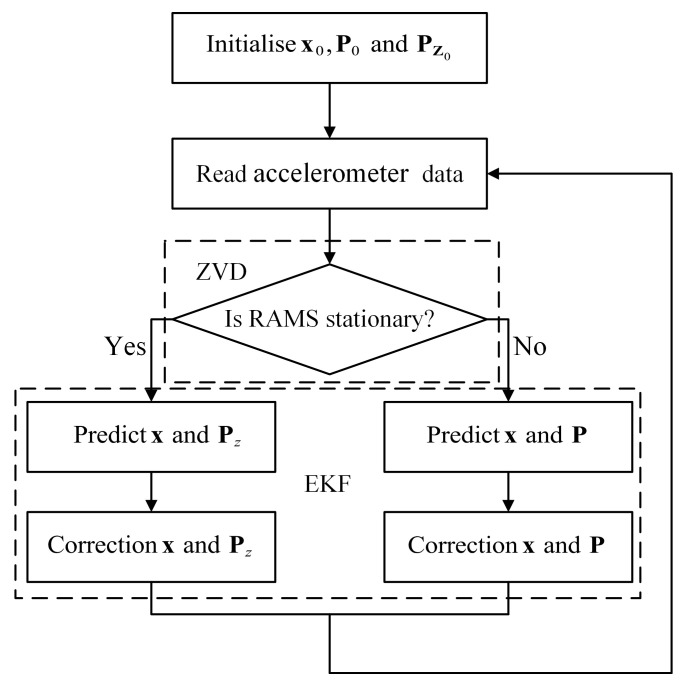
Complete procedure of rotation angle estimation.

The ZVD is still used to distinguish whether the sensor is stationary or in motion. Figure 8 shows the complete rotation angle estimation procedure.

**Figure 9:**
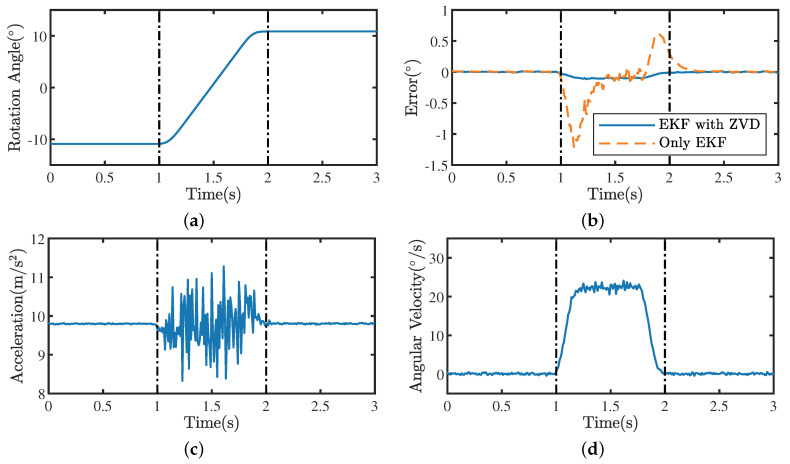
An example of the EKF with ZVD and the EKF applied to the data recorded by the RAMS. (**a**) Reference rotation angles obtained by a precision turntable. (**b**) Angle estimation errors of the EKF with ZVD (solid lines) and the EKF (dashed lines) with respect to the reference angles. (**c**) Magnitudes of the acceleration. (**d**) Magnitudes of the angular velocity.

Figure 9 shows an example of how the EFK with ZVD and the standard EKF work on data recorded by the RAMS, respectively. The reference angle in Figure 9a was obtained by a precision turntable. The error in Figure 9b is defined as the difference between the angle $\theta_{k}$ estimated using different algorithms and the reference angle ${\hat{\theta }}_{k}$:

(34) $$\mathrm{error}=\theta_{k}-{\hat{\theta }}_{k}.$$

The complete motion process in Figure 9 is divided into three parts: stationary period 1 (0–1 s), moving period (1–2 s), and stationary period 2 (2–3 s). In stationary period 1, the two algorithms produced almost the same results (see Figure 9b). In the moving period, $\left|\right|{}^{B}a_{m}\left|\right|\neq g$ and $\left|\right|{}^{B}\omega_{m}\left|\right|\neq 0$, the angle estimation error of the standard EKF was higher than that of the EKF with ZVD. In stationary period 2, the RAMS was stationary again, and the estimation error of the EKF with ZVD converged rapidly. Therefore, we can conclude that the improved EKF algorithm in this paper has significant advantages in dynamic processes.

## 4. System Design

**Figure 10:**
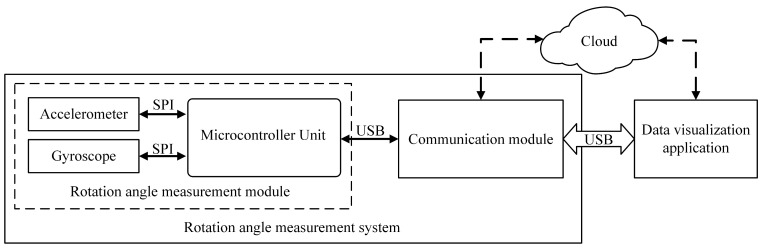
Rotation angle measurement system architecture.

The RAMS that was designed in this paper consists of a rotation angle measurement module and a 5G mobile communication module. The block diagram for the system is shown in Figure 10. The RAMS transfers the measurement results to the cloud server via 5G network. The data visualization application obtains the measurement data from the cloud server and visualizes them. Furthermore, the data visualization application can access RAMS measurements directly via USB. More details of the rotation angle measurement module are as follows.

The rotation angle measurement module (see Figure 10) is the core of the measurement in RAMS, which includes a tri-axis accelerometer (ADXL355), an IMU (BMI088) containing a tri-axis gyroscope, and a microcontroller (STM32F411). The ADXL355 [27] is a low noise density, low offset drift, low power, selectable measurement range tri-axis accelerometer with industry-leading noise, minimal offset drift over temperature, and long-term stability for precision applications with minimal calibration. The BMI088 [28] is a high-performance, low-cost inertial sensor consisting of a 16-bit digital tri-axis accelerometer and a 16-bit digital tri-axis gyroscope with high-vibration robustness and excellent temperature stability. The STM32F411 [29] is a microcontroller based on the ARM Cortex-M4 32-bit core operating frequency of up to 100 MHz. It features a single-precision floating-point unit, a full set of DSP instructions, up to five SPI interfaces (up to 50 Mbit/s), and a full-speed USB 2.0 controller.

The reason to choose two different sensors instead of having one combined one is that we need a low-noise accelerometer to achieve the measurement accuracy while saving costs. Table 2 gives the prices and accelerometer noise densities for ADXL355, ADIS16465 (a high-precision MEMS IMU IC), and BMI088, where the prices are from Digi-Key. As can be seen from the information in Table 2, the noise density of ADXL355 is much less than that of the accelerometer in BMI088, however, the price is much lower than ADIS16465. This solution can achieve the desired measurement accuracy while keeping the cost of sensors under USD 100.

The STM32F411 uses SPI to read acceleration data from ADXL355 and angular velocity data from BMI088 and uses USB to send the measurement results to the communication module. The rotation angle measurement algorithm described in Section 3 is executed on the STM32F411.

Two sensors (ADXL355 and BMI088) were separately calibrated using the method in ref. [30] to reduce the sensor measurement errors.

## 5. Simulation and Discussion

In this section, we use simulations to verify the proposed algorithm and compare it with the method in ref. [16]. The properties of the IMU used in the simulations are presented in Table 3.

### Simulation

**Figure 11:**
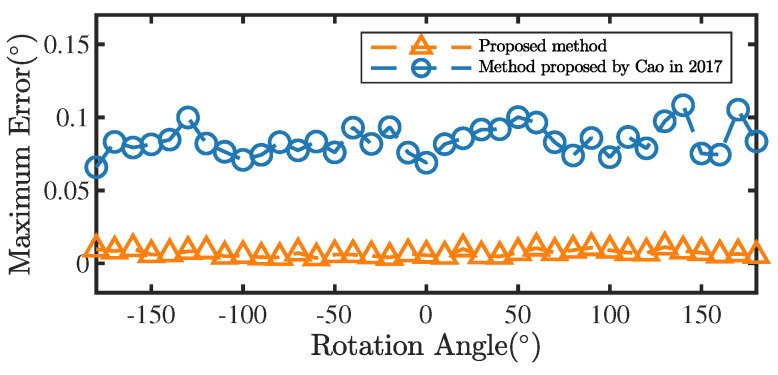
Maximum measurement errors of the two methods for different rotation angles [16].

In the simulation, we first rotated the IMU around a rotation axis and estimated the misalignment between the rotation axis and the IMU using the method in ref. [16] and the method proposed in this paper, respectively. Then, the IMU was rotated around the rotation axis from −180° to 180° and 1000 measurements calculated by the two methods were recorded at 5° intervals. Figure 11 shows the maximum measurement errors of the two methods for different rotation angles with the rotation axis ${}^{B}v={\left[\begin{array}{ccc} 0.1734 & 0.9835 & -0.0523 \end{array}\right]}^{T}$. Clearly, the measurement accuracy of the proposed rotation angle estimation method is superior against the method in ref. [16].

## 6. Discussion

Since the rotation angle measurement model in this paper relies on the rotation axis direction, the accuracy of the rotation axis direction estimation directly affects the accuracy of the measurement results.

For accelerometers, the axis misalignment, scaling factor, and fixed bias can be obtained and compensated by the method in ref. [30], while the measurement noise $n_{a}$ directly affects the accuracy of the rotational axis estimation. To reduce the effect of $n_{a}$, we should extend the stationary time as long as possible and average the acceleration data recorded during the stationary period.

**Figure 12:**
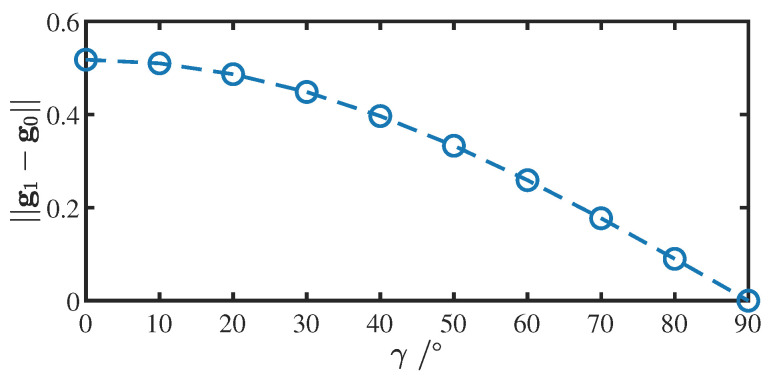
Relationship between the variation of β (angle between the rotational axis and the horizontal plane) and $\left|\right|g_{1}-g_{0}\left|\right|\left(1g=9.8\;{m/s}^{2}\right)$.

In addition, the angle between the rotation axis and the horizontal plane also has an impact on the accuracy of the rotation axis direction estimation. As the rotation axis changes from horizontal to vertical, the noise weight of ${}^{B}{\bar{a}}_{1}-{}^{B}{\bar{a}}_{0}$ in Equation (23) becomes larger. Assume that γ is the angle between the rotational axis ${}^{B}v$ and the horizontal plane, $g_{0}$ and $g_{1}$ are the ideal values of the measured acceleration of gravity for two different rotational positions ($g_{1}=R{\left(30{}^{\circ},{}^{B}v\right)}^{T}g_{0}$). The relationship between the variation of γ and $\left|\right|g_{1}-g_{0}\left|\right|$ is shown in Figure 12. When the rotation axis is vertical, rotation about this axis does not cause a change in the measured acceleration (regardless of noise). When γ=60°, the magnitude of $\left|\right|g_{1}-g_{0}\left|\right|$ is half of that at γ=0°, so in the actual measurement process, the angle between the rotation axis and the horizontal plane should preferably be less than 60°.

## 7. Experiments Result

We used a precision three-axis turntable (SGT320E from China Aviation Industry Corporation) as an experimental platform to provide the actual reference data of rotation angles. The angular position accuracy of the SGT320E is $\pm 5^{\prime \prime }$. The RAMS was compared with the method in ref. [16] and a high-precision commercial AHRS (Ellipse2-N from SBG). The estimates of the rotation angles obtained by the method in ref. [16] were calculated using raw data from the RAMS. The Ellipse2-N is a small, high-precision AHRS that contains a tri-axis gyroscope, accelerometer, and magnetometer, with a measurement accuracy of 0.1° for roll and pitch, and 0.8° for yaw.

To study the RAMS’s performance in different mounting positions, we adjusted the RAMS to different positions for several tests, taking the rotation axis direction close to the RAMS’s X axis direction (Test 1), Y axis direction (Test 2), and a tilt direction (Test 3) as examples.

All tests were divided into two steps. The first step controlled the specified rotation axis of the SGT320E to rotate three times to estimate the rotation axis direction. The second step was to control the rotation axis from −180° to 180° with a 1 s pause at 10° intervals.

The rotation axis direction estimated by the RAMS is shown in Table 4. The results were retained to four decimal places. A total of four sets of rotation angle data were obtained, corresponding to the reference rotation angles obtained by the SGT320E, the RAMS measured angles, the method in ref. [16] measured angles, and the Ellipse2-N measured angles. The Ellipse2-N measurement results were obtained by converting the measured orientations into axis-angle form. The measurement errors were computed as the difference between measured values and the reference values.

**Figure 13:**
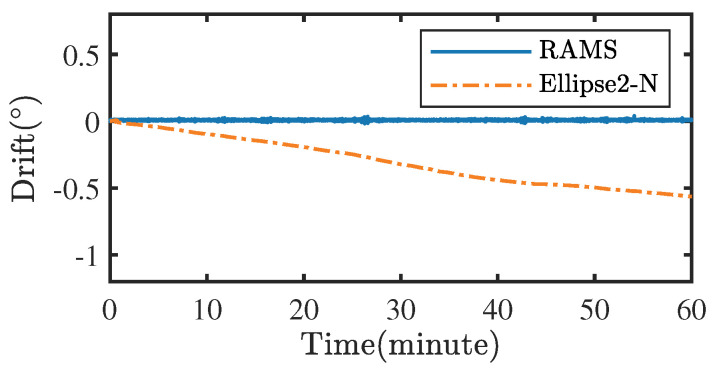
Drift of the Ellipse2-N and RAMS were left to stand for one minute.

It is common to quantify sensor performance as the static and dynamic root-mean-square error (RMSEs). The ZVD determined whether the RAMS was stationary or moving. The RMSEs of the angular measurements at different positions are summarized in Table 5, where each value is retained to four decimal places. In all three sets of tests, the static RMSEs of the RAMS are less than 0.02°, and the moving RMSEs are less than 0.5°. The static RMSEs of the method in ref. [16] are less than 0.1°, however, this method cannot obtain accurate measurements under dynamic conditions. Both the static and moving RMSEs of Ellipse2-N exceed 0.1° because the magnetometer could not effectively suppress the cumulative error of the gyroscope at the heading angle due to the frequent changes in the magnetic field in the experimental environment. After running Ellipse2-N and the RAMS for 10 min, they were left to stand still for 1 min. During this minute, the RAMS’s measurement results were held constant while the Ellipse2-N’s measurement results shifted (see Figure 13), which shows that the designed RAMS is not affected by the gyroscope integration error.

**Figure 14:**
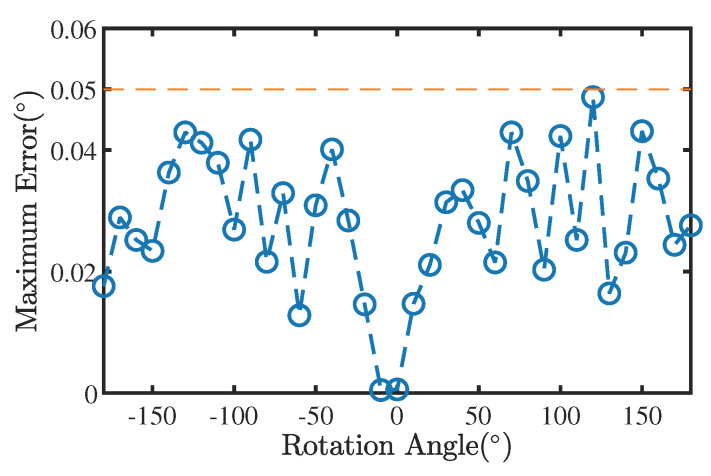
Maximum measurement error of RAMS for various angles.

The maximum measurement error for different angles is also an important indicator of system performance. As shown in Figure 14, the maximum measurement error of RAMS for various angles did not exceed 0.05°.

## 8. Conclusions

It is an important part of aircraft ground testing to accurately measure the rotation angle of movable surfaces. For existing IMU-based attitude sensors, the only way to obtain the desired measurement accuracy is to align the sensitive axis of the sensor with the movable surface rotation axis or calibrate the mounting error using precision equipment. Considering the fact that mounting errors are inevitable in the actual measurement process, this paper focuses on proposing a new measurement algorithm for rotational angle measurement without any additional calibration equipment.

The proposed algorithm uses a unit vector in the IMU frame of reference to represent the direction of the rotation axis, which can be estimated using only the stationary acceleration measurements for three different rotational positions. The process of the rotation axis direction estimation can be performed before or during the angle measurement (estimating the rotation axis direction during the measurement process requires saving the raw data from the RAMS and outputting the measurement results in real-time only after the rotation axis is estimated). The rotation angle estimation part of the proposed algorithm uses an adaptive EKF, whose correction model is built relying on the estimated rotation axis direction. The results of simulations and real experiments show that the static measurement accuracy of RAMS in the range of −180°–180° is better than that of a commercial AHRS and another measurement method in ref. [16], and the maximum static measurement error is less than 0.05°.

In addition, the RAMS designed in this paper is not only applicable to aircraft movable surface measurement but can also be applied to any rotation angle measurement project where the rotation axis is fixed, such as the measurement of a robot arm joint rotation angle and the calibration of a low-cost rotary encoder.

## Figures and Tables

**Table 1:** Parameter values of zero-velocity detection.

Parameter	Value
$th_{a_{\max}}$	9.84 $m/s^{2}$
$th_{a_{\min}}$	9.76 $m/s^{2}$
$th_{\zeta_{a}}$	0.001 $m^{2}/s^{4}$
$t_{w}$	0.01 s
$th_{t}$	0.01 s

**Table 2:** Accelerometer noise density prices for different sensor ICs.

IC	Noise Density $\left(\mu g/\sqrt{\mathrm{Hz}}\right)$	Unit Price (USD)
**ADXL355**	**22.5**	**52.14**
ADIS16465	23	798.14
BMI088	175	28.75

**Table 3:** Properties of the IMU used in the simulations.

	Accelerometer	Gyroscope
Bias	50 mg	0.7°/s
Noise Density	50 $\mu g/\sqrt{\mathrm{Hz}}$	0.02°$/s/\sqrt{\mathrm{Hz}}$
Random Walk	10 $\mu g/\sqrt{\mathrm{Hz}}$	0.014°$\sqrt{\mathrm{Hz}/s}$

**Table 4:** Rotation axis directions estimated by RAMS.

	Rotation Axis Direction
Test 1	${}^{B}v_{1}={\left[\begin{array}{ccc} 0.9932 & -0.1044 & 0.0523 \end{array}\right]}^{T}$
Test 2	${}^{B}v_{2}={\left[\begin{array}{ccc} 0.1043 & 0.9921 & -0.0698 \end{array}\right]}^{T}$
Test 3	${}^{B}v_{3}={\left[\begin{array}{ccc} 0.8070 & -0.5864 & 0.0698 \end{array}\right]}^{T}$

**Table 5:** Static and dynamic RMSE of Ellipse2-N and RAMS.

		RMSE Static (°)	RMSE Dynamic (°)
${}^{B}v_{1}$	RAMS	**0.0145**	0.4138
	Ellipse2-N	0.1091	0.3545
	Method in ref. [16]	0.0672	3.6057
${}^{B}v_{2}$	RAMS	**0.0182**	0.4133
	Ellipse2-N	0.1734	0.2624
	Method in ref. [16]	0.0804	2.7650
${}^{B}v_{3}$	RAMS	**0.0118**	0.4611
	Ellipse2-N	0.0827	0.1299
	Method in ref. [16]	0.0709	3.6634

## Data Availability

Not applicable.

## Appendix A

**Algorithm A1** Rotation Axis Direction Estimation
Input: New acceleration data ${}^{B}a$, rotation axis direction ${}^{B}v$ Output: Whether ${}^{B}v$ is successfully calculated 1: Create a data buffer Buf 2: flag←0, count←0, i←0 3: ${}^{B}\bar{a}\left[0\right]$, ${}^{B}\bar{a}\left[1\right]$, ${}^{B}\bar{a}\left[2\right]\leftarrow {\left[\begin{array}{ccc} 0 & 0 & 0 \end{array}\right]}^{T}$ 4: **if** count<3 **then** 5: **if** Zero-velocity detection result is stationary **then** 6: $Buf\left[i\right]\leftarrow {}^{B}a$ 7: flag←1 8: **else** 9: **if** flag=1 **then** 10: ${}^{B}\bar{a}\left[count\right]\leftarrow$ average the data in Buf 11: flag←0, clear Buf, i←0 12: count=count+1 13: **end if** 14: **end if** 15: i=i+1 16: **end if** 17: **if** count=3 **then** 18: ${}^{B}v\leftarrow$ calculate (15) using ${}^{B}\bar{a}\left[0\right]$, ${}^{B}\bar{a}\left[1\right]$, and ${}^{B}\bar{a}\left[2\right]$ 19: **return true** 20: **else** 21: **return false** 22: **end if**
